# Economic fluctuations and urban-rural differences in educational inequalities in mortality in the Baltic countries and Finland in 2000–2015: a register-based study

**DOI:** 10.1186/s12939-020-01347-5

**Published:** 2020-12-17

**Authors:** M. Leinsalu, A. Baburin, D. Jasilionis, J. Krumins, P. Martikainen, A. Stickley

**Affiliations:** 1 Stockholm Centre for Health and Social Change (SCOHOST), Södertörn University, 141 89 Huddinge, Sweden; 2 Department of Epidemiology and Biostatistics, National Institute for Health Development, Tallinn, Estonia; 3 Max Planck Institute for Demographic Research, Rostock, Germany; 4 Demographic Research Centre, Vytautas Magnus University, Kaunas, Lithuania; 5 Demography unit, Faculty of Business, Management and Economics, University of Latvia, Riga, Latvia; 6 Centre for Health Equity Studies (CHESS), Stockholm University and Karolinska Institutet, Stockholm, Sweden; 7 Population Research Unit, University of Helsinki, Helsinki, Finland

**Keywords:** Educational inequalities, Macroeconomic changes, Mortality, Urban-rural differences

## Abstract

We examined urban-rural differences in educational inequalities in mortality in the Baltic countries (Estonia, Latvia, Lithuania) and Finland in the context of macroeconomic changes. Educational inequalities among 30–74 year olds were examined in 2000–2003, 2004–2007, 2008–2011 and 2012–2015 using census-linked longitudinal mortality data. We estimated age-standardized mortality rates and the relative and slope index of inequality. Overall mortality rates were larger in rural areas except among Finnish women. Relative educational inequalities in mortality were often larger in urban areas among men but in rural areas among women. Absolute inequalities were mostly larger in rural areas excepting Finnish men. Between 2000–2003 and 2012–2015 relative inequalities increased in most countries while absolute inequalities decreased except in Lithuania. In the Baltic countries the changes in both relative and absolute inequalities tended to be more favorable in urban areas; in Finland they were more favorable in rural areas. The overall pattern changed during the reccessionary period from 2004–2007 to 2008–2011 when relative inequalities often diminished or the increase slowed, while the decrease in absolute inequalities accelerated with larger improvements observed in urban areas. Despite substantial progress in reducing overall mortality rates in both urban and rural areas in all countries, low educated men and women in rural areas in the Baltic countries are becoming increasingly disadvantaged in terms of mortality reduction.

## Introduction

Educational inequalities in mortality persist in Europe although in most countries mortality rates have also declined rapidly among the low educated [1]. Despite extensive research on educational inequalities in mortality, very little is known about how these inequalities differ between urban and rural residents within countries. An urban-rural mortality gap has developed in many countries over recent decades, mostly because of larger mortality reductions in urban areas [2]. Socioeconomic deprivation, more limited access to health care and unhealthy lifestyles have been related to the rural mortality disadvantage [2]. Social determinants of health are linked to wider macrolevel processes [3] that may affect urban and rural areas differently. Strong economic growth in Europe between 2000 and 2008 was more pronounced in urban areas with capital metro regions experiencing the highest per capita GDP growth rates. However, these same areas were also most negatively affected by the recession after 2008 and experienced sharper contractions in employment [4]. Some earlier studies found that overall mortality increases when the economy is expanding and falls during recessions, however, there is also some evidence that this pro-cyclical association may be related to urban areas only [5].

The impact of economic fluctuations on educational inequalities in mortality in urban and rural areas has not yet been studied. We aimed to examine urban-rural differences in educational inequalities in mortality in the Baltic countries – Estonia, Latvia and Lithuania in relation to large-scale macroeconomic changes between 2000 and 2015, while using neighboring Finland, a wealthier Nordic welfare state as a point of reference. The Baltic countries experienced rapid economic growth and low unemployment until 2008 and a deep recession with unemployment tripling afterwards; in Finland, the changes were less extreme (Supplementary Fig. 1).

## Methods

Data for Estonia, Latvia and Lithuania come from longitudinal mortality follow-up studies of population censuses in 2000/01 and 2011 where all permanent residents who participated in the census were followed from the census date until the date of death or emigration, or until the end of the follow-up period i.e. either 31.12.2011 (for the 2000/01 census) or 31.12.2015 (2011 census). The censuses in the Baltic countries combined traditional survey-based enumeration and register-based enumeration. The share of the population covered when using only survey-based enumeration varied from 91% in Latvia to 98% in Estonia [6]. The register-based data did not contain information about educational level and were thus excluded from the analysis. A sensitivity analysis performed for Latvia showed that by excluding register-based records we slightly underestimated overall mortality but that the effect on changes between the periods was minimal (Supplementary Table 1). Death data were linked from national mortality registries with 95–98% of deaths being successfully matched to census records. All data linkages were performed by National Statistical Offices. Data for Finland were obtained from the longitudinal register-based population data file of Statistics Finland covering the permanently resident total population. Data were organized into four sub-periods representing moderate economic growth (2000–2003), economic expansion (2004–2007), recession (2008–2011), and economic stabilization (2012–2015). Sociodemographic characteristics are census based and were coded using a common study protocol. Urban-rural residence was defined using national administrative classifications. In the Baltic countries, settlements with more than 2000 inhabitants (3000 in Lithuania) are considered urban. In Finland, an urban settlement is defined as a cluster of dwellings with at least 200 inhabitants per 250 × 250 square meters. Educational level was categorized as *low,* referring to the International Standard Classification of Education 2011 categories 0–2, *middle* (3–4), and *high* (5–8). Less than 1% of the values were missing for education and these cases were additionally omitted from the analyses. The analysis was restricted to the 30–74 age group to ensure that the full educational history of the subjects was covered.

Age-standardized mortality rates (ASMRs) per 100,000 person years were calculated using 5-year age groups and the 1976 European Standard Population. Educational inequalities in mortality were assessed using the relative index of inequality (RII) and the slope index of inequality (SII) [7]. The RII is a regression-based measure that adjusts the relative position of each educational group to its share in the population thus taking into account differences in the educational distribution between populations and between urban and rural areas. The SII measures absolute mortality rate differences between the lowest and highest end of the educational hierarchy. The SIIs were calculated from the RIIs and overall ASMRs using the formula SII = 2*ASMR*(RII - 1)/(RII + 1) [8].

Statistical analyses were performed using SPSS Statistics for Windows, version 26.0 (IBM Corp. 2019) and STATA 14.2 (Stata Corp., College Station, Texas, USA).

## Results

This study included approximately 889,000 deaths and 104 million person years. The percentage of low educated was larger in rural areas in all countries but the differences were less marked in Finland where the size of the urban population was also larger (Supplementary Table 2).

The ASMRs were higher in rural areas in all countries except for women in Finland where they were about the same as in urban areas (Tables 1 and 2). Between 2000–2003 and 2012–2015, the ASMRs decreased in all countries with a slightly larger decline observed in urban areas, excepting Latvian men and Finnish women where ASMRs declined somewhat more in rural areas. Although the ASMRs mostly decreased or remained the same between 2000–2003 and 2004–2007, they grew sharply in Lithuania with a larger increase occurring in rural areas. From 2004–2007 to 2008–2011, the ASMR decline accelerated in nearly all countries and was larger in urban areas. From 2008–2011 to 2012–2015 the ASMRs continued to fall with a larger decline seen more often in rural areas.

**Table 1:** Age-standardized mortality rates and educational inequalities in mortality in urban and rural areas in 2000–2015 among men in the 30–74 age group

Country	Period	Urban						Rural					
			Change		Change		Change		Change		Change		Change
		ASMR (95% CI)	%	RII (95% CI)	%	SII	%	ASMR (95% CI)	%	RII (95% CI)	%	SII	%
Finland	2000–2003	832 (824–840)	–	2.74 (2.64–2.86)	–	775	–	891 (875–908)	–	2.28 (2.09–2.49)	–	696	–
	2004–2007	778 (771–786)	−6.5	2.78 (2.68–2.89)	1.5	734	−5.3	837 (821–854)	−6.1	2.27 (2.09–2.47)	−0.3	651	−6.5
	2008–2011	710 (703–717)	−8.8	2.81 (2.71–2.92)	1.0	675	−8.0	768 (752–784)	−8.2	2.39 (2.20–2.59)	5.0	630	−3.2
	2012–2015	622 (616–629)	−12.3	2.97 (2.86–3.09)	5.7	618	−8.4	676 (662–691)	−12.0	2.14 (1.97–2.31)	−10.6	490	−22.2
			**−25.2**		**8.4**		**−20.3**		**−24.1**		**−6.4**		**−29.6**
Estonia	2000–2003	1640 (1612–1668)	–	2.97 (2.78–3.17)	–	1627	–	1792 (1755–1829)	–	2.69 (2.46–2.94)	–	1640	–
	2004–2007	1505 (1479–1531)	−8.2	2.94 (2.76–3.14)	−0.8	1484	−8.8	1694 (1658–1729)	−5.5	2.88 (2.65–3.14)	7.2	1643	0.2
	2008–2011	1217 (1194–1241)	−19.1	2.75 (2.57–2.95)	−6.5	1137	−23.4	1413 (1380–1445)	−16.6	2.95 (2.69–3.23)	2.3	1395	−15.1
	2012–2015	1071 (1049–1094)	−12.0	3.04 (2.81–3.29)	10.5	1083	−4.7	1174 (1146–1202)	−16.9	3.19 (2.90–3.50)	8.0	1226	−12.1
			**−34.7**		**2.5**		**−33.4**		**−34.5**		**18.5**		**−25.2**
Latvia	2000–2003	1701 (1679–1723)	–	2.65 (2.53–2.79)	–	1539	–	2013 (1980–2047)	–	2.40 (2.23–2.58)	–	1657	–
	2004–2007	1725 (1703–1746)	1.4	2.87 (2.74–3.02)	8.3	1668	8.4	2045 (2012–2078)	1.6	2.42 (2.25–2.59)	0.7	1695	2.3
	2008–2011	1461 (1441–1481)	−15.3	2.86 (2.72–3.02)	−0.4	1408	−15.6	1767 (1736–1798)	−13.6	2.37 (2.21–2.55)	−1.8	1438	−15.2
	2012–2015	1314 (1295–1334)	−10.0	3.25 (3.06–3.46)	13.8	1393	−1.1	1515 (1486–1543)	−14.3	2.79 (2.58–3.01)	17.6	1430	−0.6
			**−22.7**		**22.7**		**−9.5**		**− 24.8**		**16.2**		**−13.7**
Lithuania	2001–2003	1270 (1252–1288)	–	2.53 (2.39–2.67)	–	1100	–	1683 (1655–1711)	–	2.32 (2.15–2.51)	–	1339	–
	2004–2007	1524 (1508–1540)	20.0	2.67 (2.56–2.79)	5.6	1388	26.2	2090 (2064–2116)	24.2	2.55 (2.41–2.70)	9.7	1823	36.1
	2008–2011	1331 (1316–1346)	−12.7	2.79 (2.67–2.92)	4.3	1256	−9.5	1842 (1816–1867)	−11.9	2.87 (2.70–3.04)	12.6	1778	−2.5
	2012–2015	1141 (1127–1156)	−14.3	3.28 (3.11–3.45)	17.5	1215	−3.3	1560 (1537–1583)	−15.3	3.18 (2.99–3.38)	10.8	1626	−8.5
			**−10.1**		**29.5**		**10.5**		**−7.3**		**36.9**		**21.4**

Change (%) between 2000–2003 and 2012–2015 is marked in bold font, in all other cases the change is calculated in comparison with the preceding period

*ASMR* Age-standardized mortality rate per 100,000 person years, *CI* Confidence interval

*RII* Relative index of inequality, *SII* Slope index of inequality per 100,000 person years

**Table 2:** Age-standardized mortality rates and educational inequalities in mortality in urban and rural areas in 2000–2015 among women in the 30–74 age group

Country	Period	Urban						Rural					
			Change		Change		Change		Change		Change		Change
		ASMR (95% CI)	%	RII (95% CI)	%	SII	%	ASMR (95% CI)	%	RII (95% CI)	%	SII	%
Finland	2000–2003	372 (367–377)	–	2.38 (2.24–2.53)	–	304	–	377 (366–388)	–	2.49 (2.17–2.86)	–	322	–
	2004–2007	347 (342–352)	−6.8	2.49 (2.35–2.64)	4.9	297	−2.3	349 (338–359)	−7.6	2.53 (2.21–2.90)	1.4	302	−6.2
	2008–2011	323 (319–328)	−6.8	2.47 (2.34–2.61)	−1.0	274	−7.7	329 (319–340)	−5.5	2.43 (2.13–2.77)	−3.9	274	−9.3
	2012–2015	301 (296–305)	−7.0	3.07 (2.90–3.24)	24.1	306	11.7	300 (290–310)	−8.9	2.59 (2.27–2.95)	6.7	266	−2.9
			**−19.2**		**29.0**		**0.7**		**−20.5**		**4.0**		**−17.4**
Estonia	2000–2003	613 (598–627)	–	2.76 (2.52–3.02)	–	573	–	649 (629–669)	–	2.72 (2.39–3.11)	–	601	–
	2004–2007	526 (514–539)	−14.1	2.56 (2.34–2.81)	−7.0	462	−19.4	566 (547–584)	−12.8	2.90 (2.54–3.30)	6.4	551	−8.3
	2008–2011	435 (423–446)	−17.4	2.53 (2.30–2.79)	−1.3	377	−18.4	481 (463–498)	−15.1	2.76 (2.41–3.16)	−4.8	450	−18.3
	2012–2015	387 (376–398)	−11.0	2.70 (2.42–3.01)	6.7	355	−5.8	428 (412–444)	−10.9	3.08 (2.67–3.56)	11.7	437	−2.9
			**−36.8**		**−2.1**		**−38.0**		**−34.1**		**13.3**		**−27.3**
Latvia	2000–2003	656 (645–667)	–	2.64 (2.47–2.82)	–	592	–	739 (720–757)	–	2.52 (2.27–2.81)	–	639	–
	2004–2007	642 (631–652)	−2.2	2.54 (2.38–2.71)	−3.8	558	−5.7	739 (722–757)	0.1	2.50 (2.26–2.77)	−0.9	634	−0.8
	2008–2011	555 (545–565)	−13.4	2.76 (2.57–2.96)	8.5	519	−7.0	648 (632–665)	−12.3	2.99 (2.69–3.32)	19.4	646	1.9
	2012–2015	498 (488–508)	−10.4	3.27 (3.01–3.56)	18.6	529	1.9	571 (555–587)	−11.9	3.48 (3.10–3.91)	16.6	633	−2.0
			**−24.1**		**23.8**		**−10.6**		**−22.6**		**38.0**		**−0.9**
Lithuania	2001–2003	474 (465–483)	–	2.19 (2.03–2.37)	–	354	–	566 (552–581)	–	2.26 (1.99–2.56)	–	437	–
	2004–2007	541 (533–550)	14.1	2.45 (2.30–2.60)	11.5	454	28.2	740 (725–754)	30.6	2.69 (2.46–2.94)	19.0	677	54.9
	2008–2011	488 (480–495)	−9.9	2.57 (2.41–2.74)	5.1	429	−5.5	665 (651–679)	−10.1	3.00 (2.73–3.29)	11.6	665	−1.8
	2012–2015	424 (417–431)	−13.0	2.99 (2.78–3.21)	16.2	423	−1.4	557 (544–570)	−16.2	3.70 (3.35–4.09)	23.3	640	−3.8
			**−10.6**		**36.2**		**19.5**		**−1.6**		**63.9**		**46.5**

Change (%) between 2000–2003 and 2012–2015 is marked in bold font, in all other cases the change is calculated in comparison with the preceding period

*ASMR* Age-standardized mortality rate per 100,000 person years, *CI* Confidence interval

*RII* Relative index of inequality, *SII* Slope index of inequality per 100,000 person years

In all settings, higher educated men and women had lower mortality compared to the lower educated (Tables 1 and 2). Among men the RIIs were larger in urban areas except in Estonia in 2008–2015 and in Lithuania in 2008–2011. Among women the RIIs were larger in rural areas except in Finland in 2008–2015, in Estonia in 2000–2003 and Latvia in 2000–2007. Between 2000–2003 and 2012–2015 the RIIs increased in most countries in both urban and rural areas excepting rural men in Finland and urban women in Estonia. The overall RII increase was particularly large in Latvia and Lithuania (especially in rural areas) but was also substantial among rural men and women in Estonia and among urban women in Finland.

Between 2000–2003 and 2004–2007, in the Baltic countries, the RIIs increased more/decreased less in rural areas except among Latvian men. In Finland the RII increase was larger in urban areas. From 2004–2007 to 2008–2011 the RIIs increase slowed or the RIIs decreased except among women in Latvia and among rural men in Lithuania and Finland; the change was generally more favorable in urban areas. In most countries, except among rural men in Finland and Lithuania and among rural women in Latvia, the largest RII increase occurred between 2008–2011 and 2012–2015; the RII increase was larger in urban areas except among men in Latvia and among women in Estonia and Lithuania where it was larger in rural areas.

The SIIs were larger in rural areas except among Finnish men and women (in 2008–2015 only). From 2000–2003 to 2012–2015 the SIIs generally decreased, or remained about the same in most countries except in Lithuania where they increased. In Estonia and Latvia (women only) the overall SII decline was larger in urban areas; in Lithuania the SII increase was larger in rural areas. In Finland, a larger decrease was observed in rural areas. In nearly all countries the SII decline accelerated between 2004–2007 and 2008–2011, especially in urban areas.

## Discussion

Socioeconomic factors often undepin educational as well as urban-rural differences in mortality [3] and they may act to amplify each other. In accordance with previous research [2], the overall mortality rates were larger in rural areas except among Finnish women. Differences between urban and rural areas in the educational distribution and socioeconomic deprivation i.e. unemployment, poverty and social exclusion [9] may possibly explain part of the urban-rural mortality gap. The risk of impoverishment and becoming unemployed is closely connected to macroeconomic changes. In the Baltic countries, the economic consequences of the recession were more pronounced in the cities where the unemployment rate increased by four times [9]. In Finland, the economic consequences were smaller and did not differ substantially between urban and rural areas. Overall mortality rates were responsive to macroeconomic changes; between 2004–2007 and 2008–2011 the mortality decline accelerated in all countries in line with a pro-cyclical mortality pattern. The mortality decline during the recession was somewhat larger in urban areas, thus supporting the findings of an earlier study [5].

Large educational inequalities in mortality were found in both urban and rural areas. Among men the relative inequalities were often larger in urban areas but among women they were larger in rural areas. Although educational inequalities in mortality can reflect differences in employment opportunities and poverty risk as well as in healthcare access and health behaviors [3] in both urban and rural areas, we can only speculate why inequalities in mortality were larger among urban men but also among rural women. Differences in the diffusion of the tobacco epidemic might help explain this urban-rural gender gap particularly in the Baltic countries. Namely, earlier research from Estonia showed that the reversal of the educational gradient in smoking was considerably delayed among women compared with men. Women in urban areas were also more likely to have ever initiated smoking compared to women in rural areas [10], which may have contributed to compressing inequalities in mortality among urban women.

Between 2000–2003 and 2012–2015 relative inequalities in mortality increased in nearly all countries while absolute inequalities mostly decreased. However, during the recession, relative inequalities in mortality decreased or the earlier increase slowed while the decrease in absolute inequalities accelerated. These results accord with an earlier study from Spain showing that all-cause mortality decreased more during the recession, especially in socioeconomically disadvantaged groups [11]. The larger contraction of the economy in urban regions during the recession [9] might therefore explain the larger urban reduction in educational inequalities in mortality. Although relative inequalities in mortality increased in nearly all settings during the economic stabilization period, the overall changes in both relative and absolute inequalities between 2000–2003 and 2012–2015 tended to be more favorable in urban areas in the Baltic countries but in rural areas in Finland.

This study had some limitations. Although our sensitivity analysis for Latvia showed that excluding register-based data from the analysis had only a minimal effect on overall mortality changes, we cannot exclude the possibility that the effect differed by educational level or urban-rural residence. Also, the different definition of urban-rural residence used in Finland may have had some impact on comparisons with the Baltic countries. However, these possible biases are unlikely to have had any major effect on our main conclusions relating to urban-rural differences in educational inequalities in mortality in the Baltic countries.

In summary, despite substantial progress in reducing overall mortality rates in both urban and rural areas in all countries, low educated men and women in rural areas in the Baltic countries are becoming increasingly disadvantaged in terms of their mortality reduction. Although policies targeting socioeconomic deprivation in rural areas may also help to diminish educational inequalities in mortality, more research is warranted to elucidate the specific mechanisms underlying the health disadvantage of lower socioeconomic groups in rural areas.

## Supplementary Information

**Additional file 1: Supplementary Table 1.** Impact of excluding register-based census records on total mortality in the 30–74 age group in Latvia, 2000–2015. **Supplementary Table 2** Characteristics of the study populations in the 30–74 age group in 2000–2015. **Supplementary Fig. 1** Macroeconomic changes in the Baltic countries and Finland in 2000–2015. Source: The World Bank Open Data 2020. https://data.worldbank.org/indicator/NY.GDP.PCAP.CD?view=chart. Accessed 10 Apr 2020.

## Data Availability

The data that support the findings of this study are available from National Statistical Offices, i.e. Statistics Estonia, Statistics Lithuania, Central Statistical Bureau of Latvia and Statistics Finland but restrictions apply on the availability of these data, which were used under license for the current study, and so are not publicly available. Data are however available from the authors upon reasonable request and with permission of the data providers.
