# Supplementary material for: Economic fluctuations and urban-rural differences in educational inequalities in mortality in the Baltic countries and Finland in 2000–2015: a register-based study
Source: Int J Equity Health. 2020 Dec 17;19:223. doi: 10.1186/s12939-020-01347-5 (PMC7745473; doi:10.1186/s12939-020-01347-5)
Supplement: Supplementary file 1 — Additional file 1: Supplementary Table 1. Impact of excluding register-based census records on total mortality in the 30–74 age group in Latvia, 2000–2015. Supplementary Table 2 Characteristics of the study populations in the 30–74 age group in 2000–2015. Supplementary Fig. 1 Macroeconomic changes in the Baltic countries and Finland in 2000–2015. Source: The World Bank Open Data 2020. https://data.worldbank.org/indicator/NY.GDP.PCAP.CD?view=chart. Accessed 10 Apr 2020. [file 12939_2020_1347_MOESM1_ESM.docx]

**Electronic Supplementary Material**

| **Supplementary Table 1.** Impact of excluding register-based census records on total mortality in the 30–74 age group in Latvia, 2000–2015 | | | | | | |
| --- | --- | --- | --- | --- | --- | --- |
|  |  |  |  |  |  |  |
| Sex | Period | Total mortality | |  |  |  |
|  |  | Census |  | Census + registry | | Difference |
|  |  | ASMR | RR (95% CI) | ASMR | RR (95% CI) | in ASMR, % |
|  |  |  |  |  |  |  |
| Men | 2000–2003 | 1814.3 | - | 1855.7 | - | -2.2 |
|  | 2004–2007 | 1854.6 | 1.02 (1.01–1.04) | 1889.6 | 1.02 (1.00–1.03) | -1.9 |
|  | 2008–2011 | 1584.6 | 0.86 (0.84–0.87) | 1599.8 | 0.85 (0.84–0.86) | -0.9 |
|  | 2012–2015 | 1444.7 | 0.91 (0.90–0.93) | 1478.7 | 0.92 (0.91–0.94) | -2.3 |
|  |  |  |  |  |  |  |
| Women | 2000–2003 | 683.7 | - | 707.5 | - | -3.4 |
|  | 2004–2007 | 677.0 | 0.98 (0.97–1.00) | 695.1 | 0.98 (0.96–0.99) | -2.6 |
|  | 2008–2011 | 589.0 | 0.87 (0.86–0.89) | 605.0 | 0.88 (0.86–0.89) | -2.6 |
|  | 2012–2015 | 534.6 | 0.90 (0.87–0.92) | 547.9 | 0.91 (0.89–0.92) | -2.4 |
| Rate ratios (RR) are adjusted for age and calculated using Poisson regression, the preceding period is the reference category (RR=1); CI, confidence interval; ASMR, age-standardized mortality rate per 100 000 person years. | | | | | | |
|  | | | | | |  |

| **Supplementary Table 2** Characteristics of the study populations in the 30–74 age group in 2000–2015 | | | | | | | | | | | | | | | | | | | | | | | | | | | | | | | | | |  |  |  |  |  |  |
| --- | --- | --- | --- | --- | --- | --- | --- | --- | --- | --- | --- | --- | --- | --- | --- | --- | --- | --- | --- | --- | --- | --- | --- | --- | --- | --- | --- | --- | --- | --- | --- | --- | --- | --- | --- | --- | --- | --- | --- |
|  | | | | | | | | | | | | | | | | | | | | |  | | | | | | |  |  |  |  |  |  |  |  |  |  |  |  |
| Sex | Country | | Period | Deaths | | Person | | Urban | |  | |  | |  | | Rural | |  | |  | | |  | | Urban | |  |  |  |  |  |  |  |  |  |  |  |  |  |
|  |  | |  |  | | years | | Educational level | | | | | |  | | Educational level | | | | | | |  | | population | |  |  |  |  |  |  |  |  |  |  |  |  |  |
|  |  | |  |  | |  | | High | | Middle | | Low | |  | | High | | Middle | | Low | | |  | |  | |  |  |  |  |  |  |  |  |  |  |  |  |  |
|  |  | |  | N | | N | | % | | % | | % | |  | | % | | % | | % | | |  | | % | |  |  |  |  |  |  |  |  |  |  |  |  |  |
| Men | Finland | | 2000–2003 | 49699 | | 5792113 | | 29.1 | | 37.2 | | 33.7 | |  | | 15.2 | | 39.1 | | 45.7 | | |  | | 81.8 | |  |  |  |  |  |  |  |  |  |  |  |  |  |
|  |  | | 2004–2007 | 48949 | | 5805170 | | 29.3 | | 40.6 | | 30.1 | |  | | 16.1 | | 42.8 | | 41.1 | | |  | | 82.6 | |  |  |  |  |  |  |  |  |  |  |  |  |  |
|  |  | | 2008–2011 | 47849 | | 5857096 | | 27.8 | | 44.8 | | 27.4 | |  | | 15.9 | | 47.3 | | 36.8 | | |  | | 83.2 | |  |  |  |  |  |  |  |  |  |  |  |  |  |
|  |  | | 2012–2015 | 46017 | | 6106322 | | 33.8 | | 42.7 | | 23.5 | |  | | 18.9 | | 49.4 | | 31.7 | | |  | | 83.8 | |  |  |  |  |  |  |  |  |  |  |  |  |  |
|  |  | |  |  | |  | |  | |  | |  | |  | |  | |  | |  | | |  | |  | |  |  |  |  |  |  |  |  |  |  |  |  |  |
|  | Estonia | | 2000–2003 | 21605 | | 1233736 | | 31.7 | | 48.8 | | 19.5 | |  | | 15.2 | | 45.2 | | 39.6 | | |  | | 62.2 | |  |  |  |  |  |  |  |  |  |  |  |  |  |
|  |  | | 2004–2007 | 21141 | | 1306910 | | 31.6 | | 51.9 | | 16.5 | |  | | 15.5 | | 48.1 | | 36.4 | | |  | | 63.0 | |  |  |  |  |  |  |  |  |  |  |  |  |  |
|  |  | | 2008–2011 | 17401 | | 1300849 | | 29.7 | | 54.8 | | 15.5 | |  | | 15.1 | | 50.0 | | 34.9 | | |  | | 64.0 | |  |  |  |  |  |  |  |  |  |  |  |  |  |
|  |  | | 2012–2015 | 15501 | | 1333700 | | 36.2 | | 50.0 | | 13.8 | |  | | 22.8 | | 49.6 | | 27.6 | | |  | | 61.1 | |  |  |  |  |  |  |  |  |  |  |  |  |  |
|  |  | |  |  | |  | |  | |  | |  | |  | |  | |  | |  | | |  | |  | |  |  |  |  |  |  |  |  |  |  |  |  |  |
|  | Latvia | | 2000–2003 | 36624 | | 1946561 | | 19.4 | | 59.6 | | 21.0 | |  | | 7.6 | | 52.4 | | 40.0 | | |  | | 65.9 | |  |  |  |  |  |  |  |  |  |  |  |  |  |
|  |  | | 2004–2007 | 38494 | | 2042925 | | 19.5 | | 62.2 | | 18.3 | |  | | 7.7 | | 55.4 | | 36.9 | | |  | | 65.9 | |  |  |  |  |  |  |  |  |  |  |  |  |  |
|  |  | | 2008–2011 | 32490 | | 2023849 | | 18.4 | | 64.0 | | 17.6 | |  | | 7.4 | | 56.9 | | 35.7 | | |  | | 65.9 | |  |  |  |  |  |  |  |  |  |  |  |  |  |
|  |  | | 2012–2015 | 28008 | | 1903683 | | 26.2 | | 63.5 | | 10.3 | |  | | 14.4 | | 63.9 | | 21.7 | | |  | | 65.9 | |  |  |  |  |  |  |  |  |  |  |  |  |  |
|  |  | |  |  | |  | |  | |  | |  | |  | |  | |  | |  | | |  | |  | |  |  |  |  |  |  |  |  |  |  |  |  |  |
|  | Lithuania | | 2001–2003 | 32772 | | 2426813 | | 21.0 | | 60.7 | | 18.3 | |  | | 7.3 | | 55.7 | | 37.0 | | |  | | 66.3 | |  |  |  |  |  |  |  |  |  |  |  |  |  |
|  |  | | 2004–2007 | 57079 | | 3409556 | | 20.9 | | 62.4 | | 16.7 | |  | | 7.3 | | 57.9 | | 34.8 | | |  | | 67.1 | |  |  |  |  |  |  |  |  |  |  |  |  |  |
|  |  | | 2008–2011 | 49793 | | 3390429 | | 20.3 | | 63.9 | | 15.8 | |  | | 7.4 | | 59.3 | | 33.3 | | |  | | 68.1 | |  |  |  |  |  |  |  |  |  |  |  |  |  |
|  |  | | 2012–2015 | 41151 | | 3072742 | | 28.2 | | 58.4 | | 13.4 | |  | | 12.5 | | 62.3 | | 25.2 | | |  | | 65.5 | |  |  |  |  |  |  |  |  |  |  |  |  |  |
|  |  | |  |  | |  | |  | |  | |  | |  | |  | |  | |  | | |  | |  | |  |  |  |  |  |  |  |  |  |  |  |  |  |
| Women | Finland | | 2000–2003 | 25073 | | 5929293 | | 32.3 | | 34.2 | | 33.5 | |  | | 20.4 | | 38.3 | | 41.3 | | |  | | 83.5 | |  |  |  |  |  |  |  |  |  |  |  |  |  |
|  |  | | 2004–2007 | 23702 | | 5904788 | | 34.2 | | 36.8 | | 29.0 | |  | | 22.6 | | 41.4 | | 36.0 | | |  | | 84.2 | |  |  |  |  |  |  |  |  |  |  |  |  |  |
|  |  | | 2008–2011 | 23397 | | 5941203 | | 33.6 | | 40.9 | | 25.5 | |  | | 23.4 | | 45.2 | | 31.4 | | |  | | 84.9 | |  |  |  |  |  |  |  |  |  |  |  |  |  |
|  |  | | 2012–2015 | 23657 | | 6169810 | | 42.8 | | 37.4 | | 19.8 | |  | | 29.6 | | 45.9 | | 24.5 | | |  | | 85.2 | |  |  |  |  |  |  |  |  |  |  |  |  |  |
|  |  | |  |  | |  | |  | |  | |  | |  | |  | |  | |  | | |  | |  | |  |  |  |  |  |  |  |  |  |  |  |  |  |
|  | Estonia | | 2000–2003 | 11778 | | 1530689 | | 39.3 | | 43.7 | | 17.0 | |  | | 23.9 | | 43.9 | | 32.2 | | |  | | 66.4 | |  |  |  |  |  |  |  |  |  |  |  |  |  |
|  |  | | 2004–2007 | 10659 | | 1607904 | | 40.7 | | 46.1 | | 13.2 | |  | | 25.2 | | 47.3 | | 27.5 | | |  | | 67.0 | |  |  |  |  |  |  |  |  |  |  |  |  |  |
|  |  | | 2008–2011 | 8941 | | 1578775 | | 40.0 | | 49.3 | | 10.7 | |  | | 25.6 | | 50.1 | | 24.3 | | |  | | 68.0 | |  |  |  |  |  |  |  |  |  |  |  |  |  |
|  |  | | 2012–2015 | 7871 | | 1553757 | | 49.3 | | 42.1 | | 8.6 | |  | | 37.0 | | 44.8 | | 18.2 | | |  | | 65.1 | |  |  |  |  |  |  |  |  |  |  |  |  |  |
|  |  | |  |  | |  | |  | |  | |  | |  | |  | |  | |  | | |  | |  | |  |  |  |  |  |  |  |  |  |  |  |  |  |
|  | Latvia | | 2000–2003 | 21369 | | 2487931 | | 21.7 | | 59.6 | | 18.7 | |  | | 11.4 | | 51.7 | | 36.9 | | |  | | 70.3 | |  |  |  |  |  |  |  |  |  |  |  |  |  |
|  |  | | 2004–2007 | 21623 | | 2585238 | | 22.7 | | 62.4 | | 14.9 | |  | | 12.1 | | 56.0 | | 31.9 | | |  | | 70.3 | |  |  |  |  |  |  |  |  |  |  |  |  |  |
|  |  | | 2008–2011 | 18628 | | 2523669 | | 22.5 | | 65.1 | | 12.4 | |  | | 12.1 | | 59.6 | | 28.3 | | |  | | 70.3 | |  |  |  |  |  |  |  |  |  |  |  |  |  |
|  |  | | 2012–2015 | 15796 | | 2330657 | | 36.8 | | 56.4 | | 6.8 | |  | | 25.5 | | 57.1 | | 17.4 | | |  | | 70.0 | |  |  |  |  |  |  |  |  |  |  |  |  |  |
|  |  | |  |  | |  | |  | |  | |  | |  | |  | |  | |  | | |  | |  | |  |  |  |  |  |  |  |  |  |  |  |  |  |
|  | Lithuania | | 2001–2003 | 17056 | | 2949919 | | 23.1 | | 60.1 | | 16.8 | |  | | 8.8 | | 53.1 | | 38.1 | | |  | | 69.3 | |  |  |  |  |  |  |  |  |  |  |  |  |  |
|  |  | | 2004–2007 | 28361 | | 4068614 | | 23.6 | | 62.3 | | 14.1 | |  | | 9.3 | | 57.3 | | 33.4 | | |  | | 70.2 | |  |  |  |  |  |  |  |  |  |  |  |  |  |
|  | |  | 2008–2011 | 25280 | | 4015691 | | 23.6 | | 64.9 | | 11.5 | |  | | 9.8 | | 61.7 | | 28.5 | | |  | | 71.4 | |  |  |  |  |  |  |  |  |  |  |  |  |  |
|  | |  | 2012–2015 | 20937 | 3661689 | | 35.7 | | 55.2 | | 9.1 | |  | | 17.7 | | 61.0 | | 21.3 | | |  | | 70.0 | |  |  |  |  |  |  |  |  |  |  |  |  |  |  |
| The follow up in the 1st period started from the census date in the Baltic countries, i.e. 31.03.2000 in Estonia, | | | | | | | | | | | | | | | | | | | | | | | | | | | | | | | | | | | | | |  |  |
| 1.03.2000 in Latvia, and 6.04.2001 in Lithuania, on all other occasions the follow up started on January 1 and ended | | | | | | | | | | | | | | | | | | | | | | | | | | | | | | | | | | | | | |  |  |
| on December 31 in the respective periods. | | | | | | | | | | | | | | | | | | | | | | | | | | | | | |  |  |  |  |  |  |  |  |  |  |


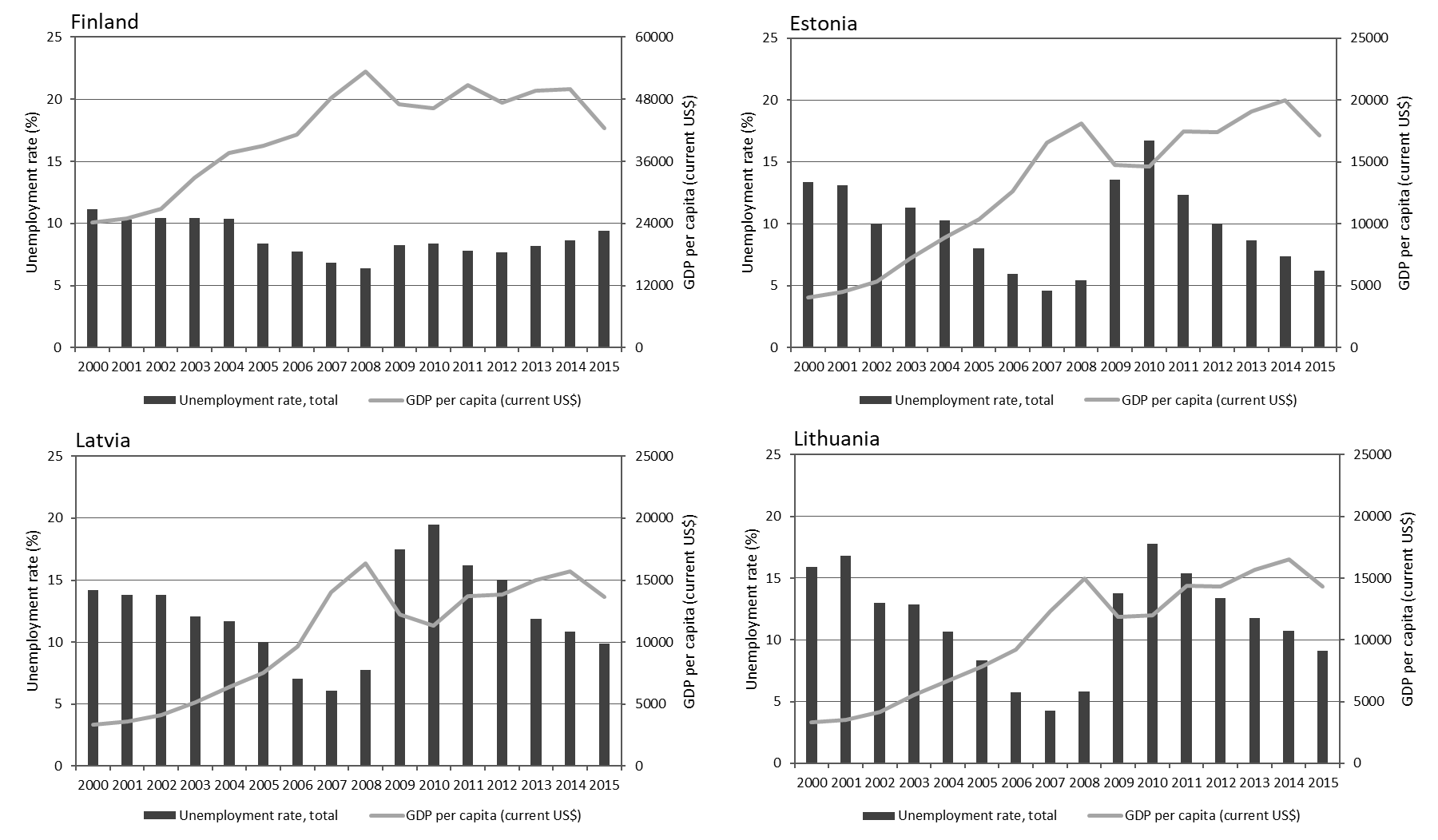


**Supplementary Fig. 1** Macroeconomic changes in the Baltic countries and Finland in 2000–2015. Source: The World Bank Open Data 2020. <https://data.worldbank.org/indicator/NY.GDP.PCAP.CD?view=chart>. Accessed 10 Apr 2020.
